# A CASE REPORT OF TREATMENT-INDUCED PULMONARY FIBROSIS IN A 68-YEAR-OLD FEMALE WITH PLATINUM-RESISTANT EPITHELIAL OVARIAN CARCINOMA.

**DOI:** 10.51894/001c.123093

**Published:** 2024-08-30

**Authors:** Rakesh Shah

**Affiliations:** 1 Internal Medicine Detroit Medical Center Sinai Grace Hospital

## 81

### INTRODUCTION

Mirvetuximab (MIRV) is the first biomarker-driven antibody-drug conjugate to target the folate receptor alpha (FRa) antigen–a protein expressed in 90% of ovarian malignancies. Clinical trials have demonstrated this drug’s efficacy in treating platinum-resistant ovarian cancer (PROC). Despite the tolerable safety profile, the drug when used with bevacizumab is associated with a rare and devastating treatment-related adverse effect of pulmonary fibrosis.

### CASE DESCRIPTION

A 68-year-old female presented to the emergency department for a one-day duration of acute onset dyspnea with cough. Medical history was significant for high-grade serous ovarian carcinoma for which the patient had undergone neoadjuvant chemotherapy, cytoreductive surgery, adjuvant chemotherapy with 10 cycles of bevacizumab, and maintenance therapy with letrozole. She was later enrolled in a phase II clinical trial with carboplatin/Mirv + Mirv maintenance with bevacizumab which she was receiving at the time of presentation. Vitals were pertinent for elevated blood pressure, tachycardia, and hypoxemia. Initial labs revealed D-dimer 7.54, and troponin 96. EKG was negative for any ischemic changes. The respiratory pathogen panel was negative for flu and COVID-19. Chest X-ray revealed predominantly basilar reticular opacities with traction bronchiectasis. CT-PE ruled out pulmonary embolism but showed worsening fibrosis.The patient’s dyspnea was treated with bronchodilators, a prolonged course of steroids, and a course of ceftriaxone. Repeat CXR re-demonstrated previously noted diffuse bilaterally prominent reticular nodular opacities. Pulmonology was consulted and they noted worsening fibrosis was likely secondary to mirvetuximab and recommended its discontinuation and a prolonged course of steroids. Infectious and cardiac workups were also unremarkable. The patient was counseled for pulmonary rehabilitation and discharged to subacute rehabilitation on oxygen therapy with the recommendation to discontinue mirvetuximab therapy permanently.

### DISCUSSION/CONCLUSIONS

In clinical studies, MIRV showed significant antitumor activity in patients with FRα-high (> 75%) PROC independent of bevacizumab exposure status. MIRV has demonstrated a tolerable safety profile with the main adverse effects including low-grade gastrointestinal and ocular symptoms. For patients with FRα levels between 25-75%, MIRV is administered in conjunction with bevacizumab for optimal efficacy. However, coadministration of both systemic agents as this case highlights increases the risk for irreversible pulmonary fibrosis and treatment failure.

